# A Method for Producing Protein Nanoparticles with Applications in Vaccines

**DOI:** 10.1371/journal.pone.0138761

**Published:** 2016-03-07

**Authors:** David S. Jones, Christopher G. Rowe, Beth Chen, Karine Reiter, Kelly M. Rausch, David L. Narum, Yimin Wu, Patrick E. Duffy

**Affiliations:** Laboratory of Malaria Immunology and Vaccinology, National Institute of Allergy and Infectious Disease, National Institutes of Health, Rockville, Maryland, 20852, United States of America; Institute of Tropical Medicine, Nagasaki University, JAPAN

## Abstract

A practical method is described for synthesizing conjugated protein nanoparticles using thioether (thiol-maleimide) cross-linking chemistry. This method fills the need for a reliable and reproducible synthesis of protein conjugate vaccines for preclinical studies, which can be adapted to produce comparable material for clinical studies. The described method appears to be generally applicable to the production of nanoparticles from a variety of soluble proteins having different structural features. Examples presented include single-component particles of the malarial antigens AMA1, CSP and Pfs25, and two component particles comprised of those antigens covalently cross-linked with the immunogenic carrier protein EPA (a detoxified form of exotoxin A from Pseudomonas aeruginosa). The average molar masses (Mw) of particles in the different preparations ranged from 487 kDa to 3,420 kDa, with hydrodynamic radii (Rh) ranging from 12.1 nm to 38.3 nm. The antigenic properties and secondary structures of the proteins within the particles appear to be largely intact, with no significant changes seen in their far UV circular dichroism spectra, or in their ability to bind conformation-dependent monoclonal antibodies. Mice vaccinated with mixed particles of Pfs25 or CSP and EPA generated significantly greater antigen-specific antibody levels compared with mice vaccinated with the respective unmodified monomeric antigens, validating the potential of antigen-EPA nanoparticles as vaccines.

## 1. Introduction

In the course of developing conjugates of plasmodial proteins as vaccines for malaria, an efficient and scalable method was developed for producing protein nanoparticles comprised of antigen alone or antigen combined with an immunogenic carrier protein (carrier).

Assembly of antigens into particles to improve their immunogenicity is an often used strategy in modern vaccine development. Nanoparticles have found applications throughout biomedicine, and vaccines in particular have benefited from structural features and other properties that can be incorporated into nanoparticles [1]. The most advanced malaria vaccine to date is a virus-like particle containing a single copy of a portion of the circumsporozoite protein (CSP) fused to a single hepatitis B surface protein molecule and mixed in a ratio of 1:4 with unfused hepatitis B surface protein molecule [2]. Self-assembled peptide nanoparticles have been shown to improve immune responses of peptide antigens [3]. The application of particle-based technologies toward vaccines has been reviewed [4].

Conjugation of antigens to protein carriers is another widely used strategy for improving vaccine potency. Polysaccharide conjugates in particular have contributed greatly to numerous effective childhood vaccines [5] [6]. Poorly immunogenic peptides and proteins can also become better immunogens when conjugated to protein carriers [7,8]. Conjugates of recombinant subunit proteins found at various stages of the malaria parasite lifecycle are being actively investigated as vaccines. Recombinant blood stage proteins AMA1 and MSP1 have been conjugated with Exoprotein A (EPA), a detoxified form of exotoxin A from *Pseudomonas aeruginosa* [9,10]. Proteins expressed in the mosquito stage (Pfs25 and Pfs28) are being investigated as vaccines for blocking malaria transmission. Conjugates of Pfs25 with EPA, OMPC (outer membrane protein complex) or with itself have been shown to be more immunogenic than the unconjugated forms [9,11,12]. Conjugation of Pfs28 to EPA also improved immunogenicity [13]. Various conjugated forms of CSP, expressed in the pre-erythrocytic stage of the parasite lifecycle, have been reported [14].

A significant impediment to developing protein conjugate vaccines has been poor yield and lack of reproducibility. Consequently, protein conjugate vaccines produced for early-stage preclinical testing have been difficult to reproduce in the quantities needed for later stages. An efficient process was needed for preparing characterizedconjugates for pre-clinical studies, which could be adapted to scale-up studies leading to the production of clinical grade material in conformance with current good manufacturing practices (cGMP), if warranted. Toward that end a process was developed for producing protein conjugates by cross-linking antigen and carrier to form conjugated protein nanoparticles of suitable size for complete biochemical and biophysical characterization and sterile filtration. This paper describes a practical synthetic method for producing soluble protein nanoparticles composed of one or two proteins. Examples include recombinant malarial antigens Pfs25, CSP and AMA1 with or without inclusion of EPA as a carrier.

## 2. Materials and Methods

### 2.1 Recombinant Proteins and Monoclonal Antibodies

AMA1 from the *P*. *falciparum* FVO malaria parasite clone (molecular weight, 61,906 Da) was expressed in *Pichia pastoris* [15]. EPA (molecular weight, 66,975 Da) was expressed in *E*. *coli* [9]. Pfs25H from the *P*. *falciparum* NF54 isolate (molecular weight 20,438 Da) was expressed in *P*. *pastoris* [16]. Recombinant Pfs25M from the *P*. *falciparum* NF54 isolate without a His6 fusion tag (molecular weight 18,712) was expressed in *P*. *pastoris* and characterized in a manner similar to Pfs25H. The *P*. *falciparum* 3D7 CSP clone, CSPM3 (molecular weight 32,578), was expressed in *P*. *pastoris* and characterized as previously described [17]. *P*. *falciparum* specific monoclonal antibodies against AMA1, identified as 4G2, and against CSPM3, identified as 1G12, that inhibit parasite development and recognize conformation-dependent epitopes have been previously reported [18,19].

### 2.2 Reagents and Buffers

N-(ɛ-maleimidocaproyloxy)succinimide (EMCS) (PubChem CID: 5091655), N-(ɛ-maleimidocaproyloxy)sulfo-succinimide sodium salt (sulfo-EMCS) (PubChem CID: 4229287), and S-acetylthioglycolic acid N-hydroxysuccinimidyl ester (SATA) (PubChem CID: 127532), were purchased from Pierce Biotechnology Inc. (Rockford, IL).

Buffers used are as follows: pH 6.5 PBSE (100 mM sodium phosphate, 150 mM NaCl, 5 mM EDTA); pH 7.2 PBSE (100 mM sodium phosphate, 150 mM NaCl, 5 mM EDTA); deacetylation buffer (0.5 M NH_2_OH^.^HCl, pH 7.2 PBSE); and PBS (1.04 mM KH_2_PO_4_, 2.97 mM Na_2_HPO_4_.7H_2_O, 154 mM NaCl, pH 7.4).

### 2.3 Methods

Protein solutions were concentrated and buffer exchanges were accomplished using centrifugal membrane filtration devices with a 10 kDa nominal molecular weight cutoff (catalogue item, Amicon Ultra-15 PLGC Ultracel-PL Membrane, 10 kDa) manufactured by Millipore Corporation (Billerica, MA). Buffer exchanges were accomplished with multiple dilutions and concentrations using the buffer of choice to achieve a 1000 fold exchange (e.g., three iterations of a tenfold dilution step followed by tenfold concentration step). Protein concentrations were calculated from UV absorbance at 280 nm using extinction coefficients derived from amino acid composition (AMA1, 1.206 mL mg^-1^; CSPM3, 0.268 mL mg^-1^; EPA, 1.299 mL mg^-1^; Pfs25H, 0.315 mL mg^-1^; Pfs25M, 0.312 mL mg^-1^) [20]. The extinction coefficients of maleimide-modified proteins were adjusted upwards by 515 L mol^-1^ per attached maleimide group. CD spectra were acquired using a J-815 CD Spectrometer (Jasco Analytical Instruments, Easton, MD). Reaction yields are reported as percentage of total protein recovered.

### 2.4 Particle Synthesis

A detailed description of the method for producing protein nanoparticles consisting of Pfs25M and EPA (Pfs25M-EPA) is described. The other protein nanoparticles were prepared similarly. All reactions were carried out by combining reactants in solution with stirring at 22°C, using a constant temperature water bath to maintain temperature.

#### 2.4.1 Thiolation of Pfs25M

To 4.6 ml of a stirred 2.07 mg/ml solution of Pfs25M (9.51 mg, 5.08 x 10^−7^ mol) in pH 7.2 PBSE, pre-equilibrated at 22°C, was added 38.2 μL of a 23 mg/mL (100 mM) solution of SATA (0.879 mg, 3.81 X 10^−6^ mol) in DMSO, and the mixture was stirred for 1 hour. The mixture was exchanged into fresh pH 7.2 PBSE, concentrated to volume of approximately 4.6 ml, and 0.4 mL of deacetylation buffer was added. The mixture was incubated for 1 hour at RT with gentle agitation, and then it was exchanged into pH 6.5 PBSE and concentrated to provide 4.1 mL of thiolated Pfs25M: linker stoichiometry, 3.2 thiols per molecule of Pfs25; concentration, 1.98 mg/mL; yield, 8.11 mg (86%).

#### 2.4.2 Maleimide modification of EPA

To 2.0 ml of a stirred 2.83 mg/ml solution of EPA (5.65 mg, 8.44 x 10^−8^ mol) in pH 7.2 PBSE, pre-equilibrated at 22ºC, was added 46.5 μL of a 30.8 mg/ml (100 mM) solution of EMCS in DMSO (1.43 mg, 4.65 x 10^−6^ mol), and the mixture was stirred for 90 min. The mixture was exchanged into pH 6.5 PBSE to provide 1.1 mL of maleimide-activated EPA: linker stoichiometry, 7.9 maleimides per molecule of EPA; concentration, 5 mg/mL; yield, 5.5 mg (97%).

#### 2.4.3 Pfs25M-EPA nanoparticles

The solution of thiolated Pfs25M (Pfs25M-SH_3.2_) in pH 6.5 PBSE was concentrated to 9.49 mg/ml, and the solution of maleimide-modified EPA (EPA-mal_7.9_) in pH 6.5 PBSE was concentrated to 11.96 mg/ml. The concentration of EPA-mal_7.9_ was adjusted such that the total protein concentration would be 5 mg/ml after adding Pfs25M-SH_3.2_. That was accomplished by adding 0.97 ml of pH 6.5 PBSE to 0.35 mL (4.2 mg, 6.25 x 10^−8^ mol) of the 11.96 mg/mL EPA-mal_7.9_ solution. The solution was equilibrated at 22°C, and 0.54 mL of the 9.49 mg/ml solution of Pfs25M-SH_3.2_ (5.1 mg, 2.73 x 10^−7^ mol) was added. The mixture was kept stirring for 1 hour, at which time 108 μL of a 0.8 mg/mL solution of cysteine hydrochloride (0.086 mg, 4.94 x 10^−7^ mol) in pH 6.5 PBSE was added, and the mixture was stirred an additional 15 minutes. Purification was accomplished using a 16 mm x 60 cm Superdex 200 column (GE Healthcare Life Sciences, Piscataway, NJ) with a flow rate of 1 ml/min using PBS as the eluent. Fractions containing particles were pooled to provide 1.90 ml of Pfs25M-EPA solution: concentration, 3.14 mg/ml; yield, 5.99 mg (64%).

### 2.5 Determination of linker stoichiometry

#### 2.5.1 Determination of thiol linkers

Thiol concentrations were determined by incubating the thiolated protein with 4,4’-dithiodipyridine (DTDP) at pH 6.5 and measuring absorbance at 324 nm [21]. The concentration of thiol groups was calculated from the absorbance using an extinction coefficient of 21,400 L M^-1^ for the 4-thiopyridone product, and the result was validated by comparing with cysteine standards included in the assay.

#### 2.5.2 Determination of maleimide linkers

Maleimide concentrations were determined by measuring consumption of thiol groups when maleimide-modified proteins were mixed with cysteine. Aliquots of the maleimide-modified protein, or buffer alone, were added to standard cysteine solutions, and the mixtures were incubated for 1 hour at room temperature. Thiol concentrations were measured as described above using DTDP. Maleimide concentration was calculated as the difference between the thiol concentration of the cysteine solution plus buffer and the cysteine solution plus maleimide-modified protein.

### 2.6 Determination of molar mass and hydrodynamic radius

The average molar mass (Mw) and hydrodynamic radius (Rh) of the conjugates were determined by size exclusion chromatography coupled with multi-angle light scattering (SEC-MALS). A Dawn Helios 18 angle light scattering detector fitted with quasi elastic light scattering (QELS) (Wyatt Technologies, Santa Barbara, CA) was used to collect light scattering signals, and an OptilabRx refractive index detector (Wyatt) was used to simultaneously measure concentration. The signals from both detectors were processed using Astra software (Wyatt) to determine Mw and Rh. The size exclusion columns used were G4000PWxl and G5000PWxl (Tosoh Bioscience, King of Prussia, PA).

### 2.7 Determination of protein composition

Amino acid analyses were performed at the W.M. Keck Biotechnology Resource Lab at the Yale School of Medicine (New Haven, CT). The molar amino acid compositions were determined for each EPA-containing nanoparticle and for the individual unmodified proteins. The molar ratios of the two proteins comprising the nanoparticles were calculated as previously described [22].

### 2.8 Vaccinations of mice

Animal protocols were carried out in compliance with National Institutes of Health guidelines and under the auspices of Animal Care and Use committee approved protocols. For Pfs25H, CSPM3 and particles containing Pfs25H, Pfs25M and CSPM3, female CD-1 mice were vaccinated on days 0 and 28 by intramuscular injection of 0.05 mL of vaccine formulation containing the doses indicated adsorbed on Alhydrogel, and the animals were bled on day 42. For AMA1 and particles containing AMA1, female mice were vaccinated on days 0, 14 and 28 by subcutaneous injection of 0.10 mL of vaccine formulation containing the doses indicated adsorbed on Alhydrogel, and the animals were bled on day 42. The percentage of protein bound to Alhydrogel in each formulation was determined to be greater than 95% (100%—free protein). Supernatants were examined by polyacrylamide gel electrophoresis with visualization by silver staining. In each case the amount of free protein was below the limit of detection (5% of load).

Animal studies were performed following the guidelines approved by the National Institutes of Health (NIH) Animal Care and Use Committee (ACUC) and approved by the National Institutes of Health Animal Care and Use committee, according to the Institutional Animal Care and Use Committee (IACUC) approved protocol. The National Institutes of Allergy and Infectious Disease (NIAID), Division of Intramural Research (DIR) Animal Care and Use Program (ACUC), as part of the NIH Intramural Research Program (IRP), complies with all applicable provisions of the Animal Welfare Act and other Federal statutes and regulations relating to animals. The Program acknowledges and accepts responsibility for the care and use of animals involved in activities covered by the NIH IRP’s PHS Assurance #A4149-01.

### 2.9 Measurement of antibody levels

Antigen-specific antibody levels were determined using standardized ELISA[10] with un-modified antigens as the plate antigens [23]. Absorbance based ELISA units (EU) were determined relative to a reference antisera obtained from mice immunized with the un-modified antigen. Statistical significance for differences between groups was tested using a Mann-Whitney test, performed with Prism software (GraphPad Software, Inc., La Jolla, CA).

### 2.10 *In vitro* parasite growth inhibition assay

Groups of five female New Zealand rabbits were vaccinated intramuscularly with 0.05 mL of formulation containing 25 μg (AMA1 content) of conjugate or monomeric AMA1 adsorbed on 800 μg of Alhydrogel. Vaccinations occurred on days 0 and 56. The animals were bled on day 70. IgG was purified from individual rabbits that had been vaccinated with conjugate AMA1-AMA1 or monomeric AMA1, and the sera were tested in a standardized parasite growth inhibition assay [24,25].

## 3. Results and Discussion

### 3.1 Preparation of protein nanoparticles

The method we describe in this communication was developed as a way to overcome shortcomings that were identified in the preparation of malarial antigen conjugates. Conjugates with the immunogenic carrier protein EPA, prepared using thioether chemistry, had been shown to be significantly more immunogenic than un-modified antigens, thus establishing conjugation as an attractive malaria vaccine strategy [9,13]. The antigens had been thiolated using N-acetylhomocysteine thiolactone as the thiolating reagent [26] and subsequently reacted with maleimide-modified EPA to form conjugates. We found this method of production to suffer from low yield and variable product composition, with significant amounts of unmodified antigen remaining. These shortcomings were attributed to low and variable degrees of thiolation.

**Fig 1:**
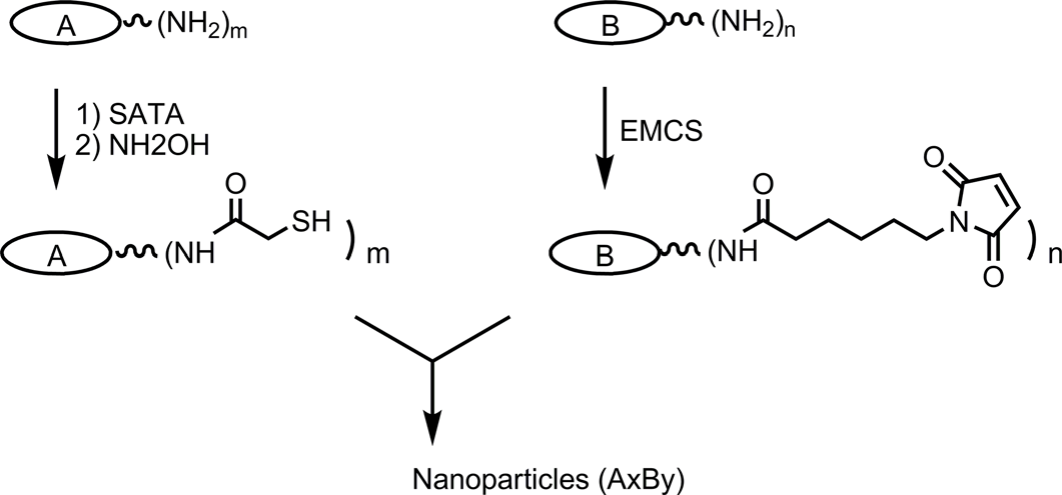
Multiple thiol or maleimide linkers are non-specifically attached to lysine amines on the protein molecules by treatment with excess SATA or EMCS. Particles composed of A and B are formed by mixing thiolated protein with maleimide-modified protein.

Our approach to improving the conjugation process was to increase the degree of antigen thiolation, which we surmised would drive the conjugation reaction toward higher molecular weight cross-linked products and thereby minimize the amount of un-conjugated protein. We used SATA as the thiolating reagent because it reacts very efficiently with lysine amino groups on proteins under mild conditions [27], and the degree of thiolation is easily controlled as a function of the concentration of SATA. Maleimide-modification of proteins was accomplished using EMCS as previously described [9,13]. SATA and EMCS are both commercially available linkers, provided as activated esters that react to form covalent amide bonds with lysine amines. After reaction with SATA, the acetyl groups are removed from the linkers using a hydroxylamine-containing buffer to generate free thiols. Fig 1 shows the stepwise process of attaching thiol and maleimide linkers to proteins and subsequent cross-linking of the two modified proteins to form particles.

Numerous test reactions were carried out to elucidate appropriate reaction parameters (data not shown). This practice is highly recommended, particularly when protein quantities are limited. The necessary concentrations of SATA or EMCS can be conveniently determined using small-scale pilot reactions with approximately 1 mg of protein. Conditions for forming particles can be determined using approximately 500 μg of protein. Protein concentration and linker stoichiometry, the average number of linkers on the proteins, were found to be key determinants of particle size (Mw and Rh). In general 3–4 linkers on each protein were found to be sufficient for optimal reaction. The relative number of linkers on each protein component also proved to be important. Larger particle sizes and higher yields were favored when the molar proportions of thiol and maleimide linkers were roughly equivalent; therefore, particles comprised of proteins with significantly different molecular weights were best prepared when the larger protein contained proportionally more linkers than the smaller one. Reactions in which thiolated protein comprised 40–55% of the protein mass appeared to result in optimal cross-linking (higher Mw and yield). The mass composition of particles comprised of two different proteins is similar to mass composition of the proteins used in the reaction.

The thiolation of Pfs25M was described in detail in the methods section. All the thiolation reactions were performed identically except the molar excess of SATA. After reaction with SATA, the mixtures were exchanged into fresh buffer to remove reagents and byproducts. Deacetylation was accomplished by treating with a hydroxylamine containing buffer. The solution was then exchanged into pH 6.5 buffer, and the number of attached thiols was determined. The conditions and results of thiolation reactions are shown in Table 1.

**Table 1:** Thiolation reaction parameters^a^.

Thiolated protein	SATA(mole equivalents)
Pfs25M-SH_3.2_	7.5
Pfs25H-SH_4.0_	20
Pfs25H-SH_2.9_	13
CSPM3-SH_3.5_	10
CSPM3-SH_3.3_	10
AMA1-SH_3.2_	20
AMA1-SH_3.3_	20

^a^ Reactions were performed as described in detail for thiolation of Pfs25M, with the exception of the number of moles of SATA used per mole of protein.

Maleimide modifications were accomplished using EMCS as described in detail in the methods section for the maleimide modification of EPA. All the maleimide modifications were performed identically except the molar excess of EMCS and the protein concentrations. After reacting the protein with EMCS, the product was exchanged in pH 6.5 buffer to remove reagents and byproducts, and the number of attached maleimides was determined. The conditions and results of maleimide modification reactions are shown in Table 2.

**Table 2:** Maleimide-modification reaction parameters^a^.

Maleimide-modified protein	EMCS(mole equivalents)	Protein concentration(mg/ml)
EPA-mal_7.9_	55	3.0
Pfs25H-mal_4.3_	30	2.0
EPA-mal_6.9_	45	3.0
CSPM3-mal_3.1_	8	2.0
EPA-mal_6.5_	40	3.0
AMA1-mal_4.7_	20	2.0
EPA-mal_6.6_	150^b^	2.0

^a^ Reactions were performed as described in detail for maleimide-modification of EPA, with the exception of the number of moles of EMCS used per mole of protein and the concentration of protein.

^b^ Sulfo-EMCS dissolved in water was used instead of EMCS.

Particles were formed by mixing the thiolated and maleimide-modified proteins at a predetermined final concentration of both proteins in pH 6.5 buffer. After one hour, excess cysteine was added to quench possible remaining maleimide groups. A detailed description of the formation of Pfs25M-EPA particles is presented in the Methods Section. The procedures used to prepare the other particles were very similar, with differences in the number of attached thiol and maleimide linkers, the molar proportions of the modified proteins, and the total protein concentration in the reaction. Those parameters and the overall yields of protein in the purified particle preparations are listed in Table 3.

**Table 3:** Particle forming reaction parameters^a^.

Particle preparation	Thiolated antigen(mole x 10^−8^)		Maleimide-modified antigen or EPA(mole x 10^−8^)		Protein^b^ concentrationmg/ml	Yield ^d^
Pfs25M-EPA	Pfs25M-SH_3.2_	(27.3)	EPA-mal_7.9_	(6.25)	5.0	58%
Pfs25H-Pfs25H	Pfs25H-SH_4.0_	(18.9)	Pfs25H-mal_4.3_	(17.6)	2.0	46%
Pfs25H-EPA	Pfs25H-SH_2.9_	(15.7)	EPA-mal_6.9_	(5.23)	10.0	38%
CSPM3-CSPM3	CSPM3-SH_3.5_	(8.33)	CSPM3-mal_3.1_	(9.40)	13.0	54%
CSPM3-EPA	CSPM3-SH_3.3_	(16.7)	EPA-mal_6.5_	(8.47)	9.6	30%
AMA1-AMA1	AMA1-SH_3.2_	(4.71)	AMA1-mal_4.7_	(3.21)	1.9	26%
AMA1-EPA	AMA1-SH_3.3_	(10.5)	EPA-mal_6.6_	(5.24)	10.2	23% +11%^c^

^a^ Reactions were performed as described in detail for Pfs25M-EPA with the exception of the concentration of protein in the reaction, number of linkers on the proteins, and the relative masses of linker-modified proteins used. Thiolated antigens were reacted with antigen or EPA modified with maleimides.

^b^ Total concentration of protein.

^c^ Two pools were collected separately from one reaction (23% yield of pool with Mw of 3,420 kDa, 11% yield of pool with Mw of 909 kDa).

^d^ Overall yield of protein, including modifications of proteins with linkers.

**Fig 2:**
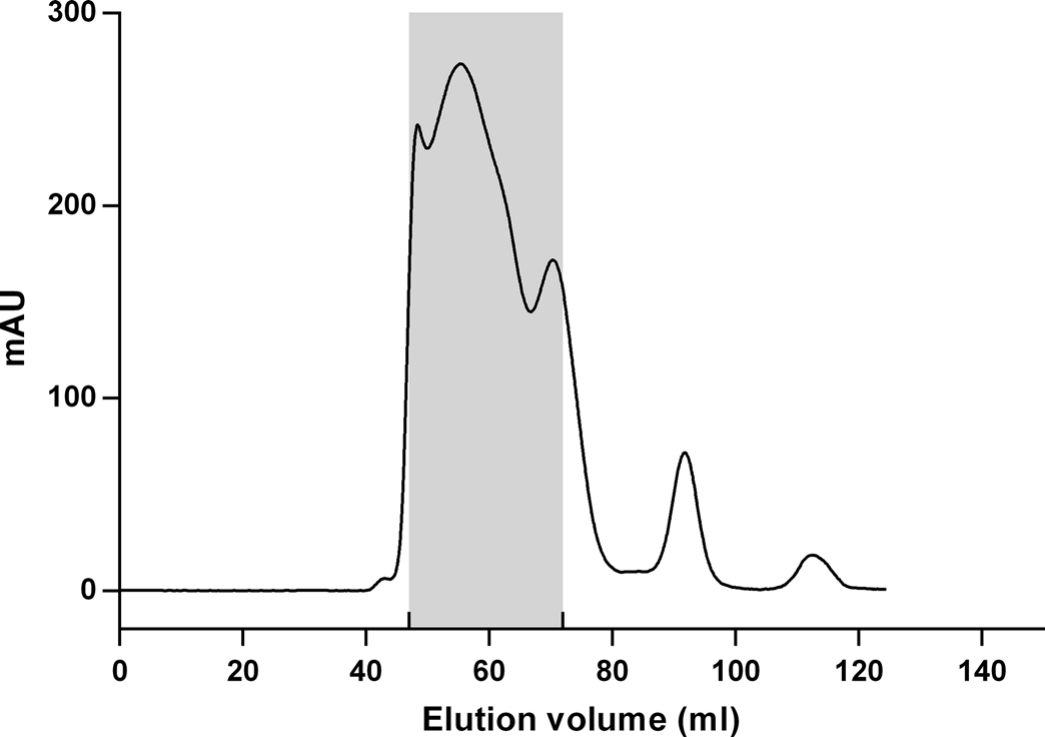
Absorbance profile (280 nm) of fractionation of Pfs25M-EPA across a 16 mm X 60 mm column pack with Superdex 200 matrix. The shaded area represents the fractions that were pooled.

**Fig 3:**
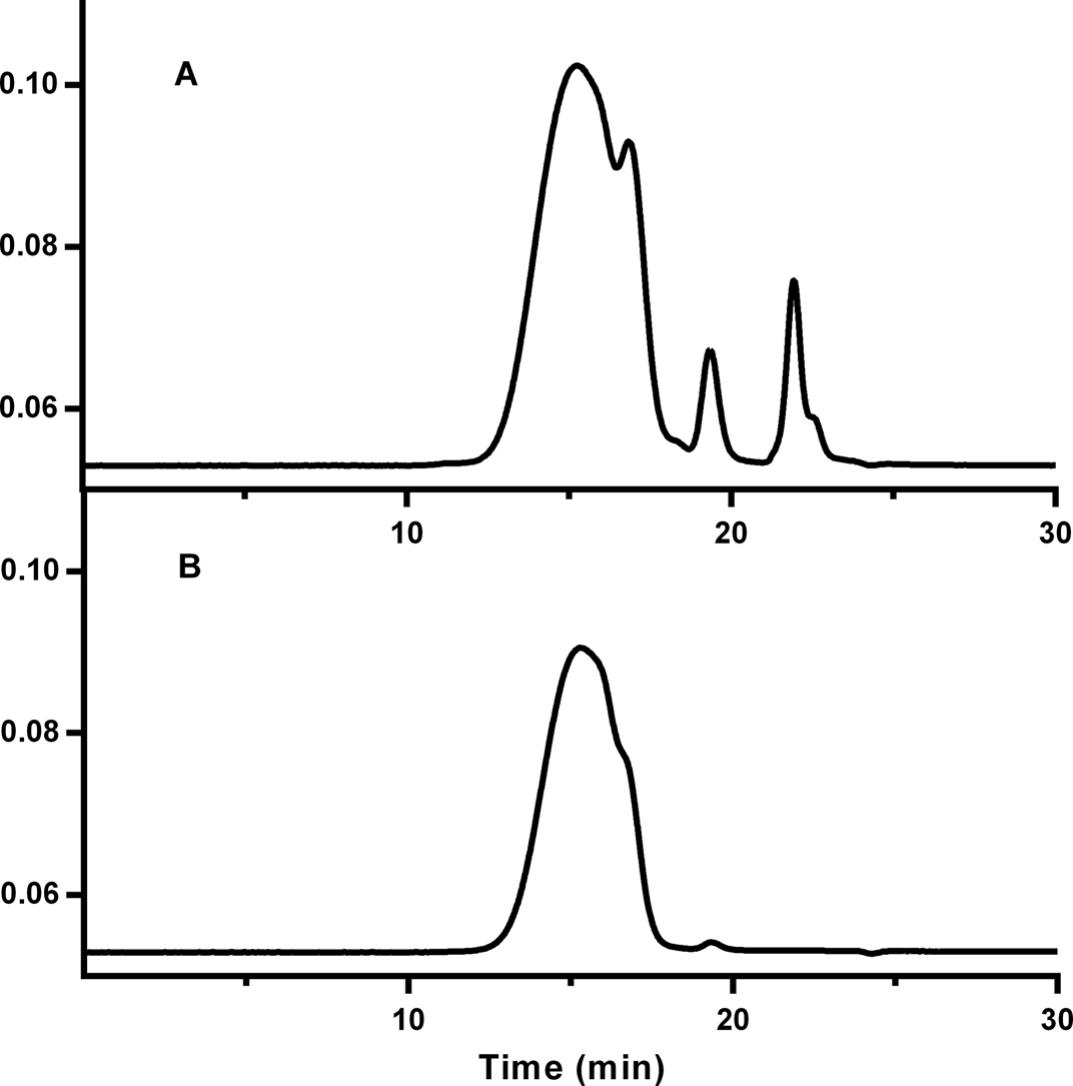
Absorbance profile (280 nm) of analytical HPLC of Pfs25M-EPA using a G5000PWxl column (Toso Biosciences); A) before purification and B) after purification. The Y axis represents detector voltage.

Purification of particles was accomplished using preparative size exclusion chromatography. The majority of UV absorbing product was collected, excluding monomeric proteins and low Mw conjugates. Decisions on what fractions to pool were made based on polyacrylamide gel electrophoresis of fractions on the low molecular weight end of the peak. The 280 nm absorbance profile of the fractionation of Pfs25M-EPA particles is shown in Fig 2. Fig 3 shows analytical SEC run on the particles before and after the fractionation process. In contrast to a standard SEC chromatographic peak of greater than 95% purity and good symmetry for example, it is accepted that asymmetrical peaks may be observed.

### 3.2 Characterization of nanoparticles

Particles were characterized with respect to size and composition. The physical parameters of average molar mass (Mw) and hydrodynamic radius (Rh) were determined from size exclusion chromatography using static and dynamic light scattering detectors. The molar composition of two-component EPA-containing particles was determined from amino acid analysis data. A summary of the particle characterizations is reported in Table 4. The larger particle size seen for CSPM3 particles is consistent with the elongated or rod-like shape reported for CSP constructs [19].

**Table 4:** Particle characterization.

Particle	Molarcomposition^a^	Mw^b^(kDa)	Rh^c^(nm)
Pfs25M-EPA	4.1:1.0	810 ± 40	14.4 ± 0.7
Pfs25H-Pfs25H	NA	487 ± 24	12.1 ± 0.7
Pfs25H-EPA	2.4:1.0	1,584 ± 80	19.0 ± 1.0
CSPM3-CSPM3	NA	1,740 ± 80	38.3 ± 1.9
CSPM3-EPA	1.7:1.0	1,331 ± 65	23.9 ± 1.2
AMA1-AMA1	NA	711 ± 35	Not measured
AMA1-EPA^d^	1.6:1.01.7:1.0	3,420 ± 150909 ± 50	27.6 ± 1.414.8 ± 0.8%

^a^ Moles of antigen per mole of EPA was determined from amino acid analysis.

^b^ Mw was calculated from multi-angle light scattering data.

^c^ Average hydrodynamic radius was calculated from dynamic light scattering data.

^d^ Two separate fractions were collected.

**Fig 4:**
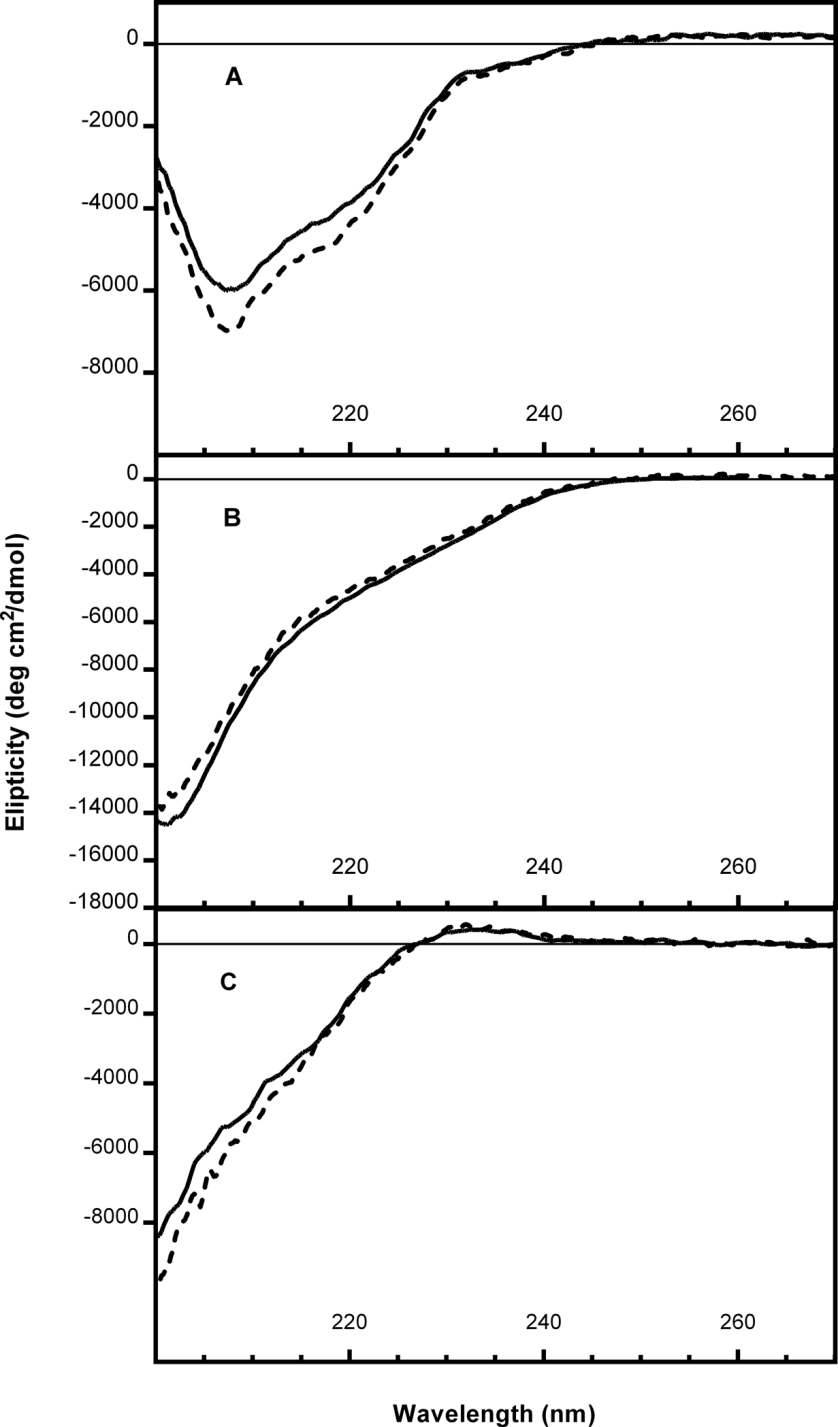
CD spectra of un-modified proteins and single component particles: A) 4.04 μM AMA1 (solid line) and 4.04 μM (AMA1 content) AMA1-AMA1 (broken line); B) 15.34 μM CSPM3 (solid line) and 6.14 μM (CSPM3 content) CSPM3-CSPM3 (broken line); C) 9.78 μM Pfs25H (solid line) and 9.78 μM (Pfs25H content) Pfs25H-Pfs25H (broken line). Stock solutions of proteins and particles in PBS were diluted with water to the stated concentrations. Spectra were obtained at 20°C.

Circular dichroism spectroscopy (CD) and binding to monoclonal antibodies were used to qualitatively compare the antigens before and after incorporation into particles. Fig 4 shows far UV optical ellipticity profiles of AMA1 vs. AMA1-AMA1 particles, CSPM3 vs. CSPM3-CSPM3 particles and Pfs25H vs. Pfs25H-Pfs25H particles. The similarity of the spectra before and after incorporation into particles indicates that the secondary structures of the antigens within the particles were largely retained [28]. AMA1-AMA1 particles and CSPM3-EPA particles were tested and shown to retain the ability to bind antigen-specific conformation-dependent monoclonal antibodies using Western transfers from polyacrylamide gels (see S1 and S2 Figs). Binding of antigen-specific conformation-dependent monoclonal antibodies to Pfs25H-EPA was previously demonstrated [29].

### 3.3 Immunogenicity of nanoparticles

**Fig 5:**
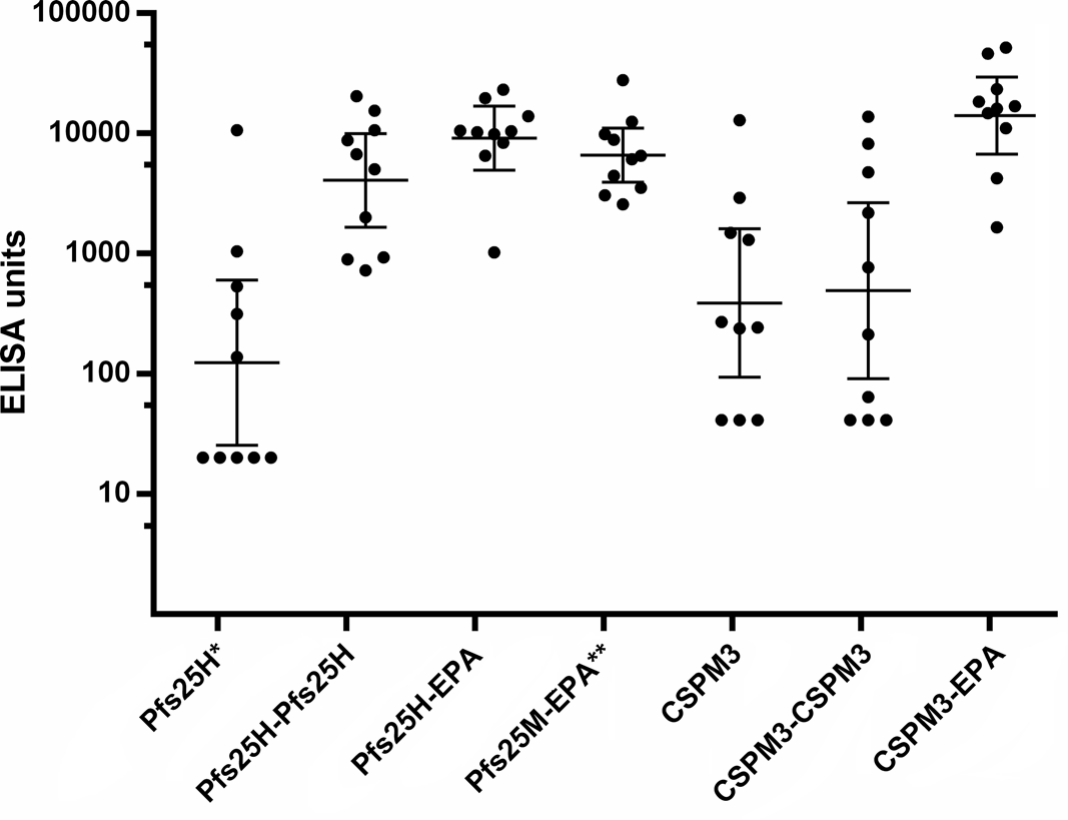
Antibody levels in mice vaccinated with un-conjugated or conjugated forms of Pfs25H, Pfs25M, and CSPM3, expressed as ELISA units (dilution sera to obtain an OD of 1). Mice were injected IM twice, 28 days apart, with 0.5 μg antigen content (* 2.5 μg for Pfs25H) of the vaccines adsorbed on 80 μg of Alhydrogel (** 45 μg of Alhydrogel for Pfs25M-EPA). Serum antibody levels were determined two weeks later by ELISA on plates coated with un-modified antigen. Dots represent ELISA units reported for individual mice. Center line represents the geometric mean, and error bars are 95% confidence levels. Statistics (Kruskal-Wallis with Dunn’s multiple comparisons): p < 0.05 for Pfs25H-EPA vs. Pfs25H; CSPM3-EPA vs. CSPM3- CSPM3; CSPM3-EPA vs. CSPM3.

Mice were vaccinated with the particles adsorbed on alum to assess antigen-specific immunity. Fig 5 shows ELISA antibody units (dilution to achieve 1 OD) for day 42 sera from mice vaccinated twice intramuscularly (days 0 and 28) with Pfs25 and CSPM3 particles, and unmodified proteins included as comparators. Antigen-specific antibody levels achieved with Pfs25 or CSPM3 particles were as high as or higher than the levels achieved with the unmodified proteins, significantly higher for the EPA containing particles. AMA1 particles and unmodified AMA1 were tested under a different protocol (subcutaneous vaccination, days 0, 14 and 28). Under these conditions AMA1, AMA1-AMA1 and AMA1-EPA generated similar levels of antigen-specific antibody (S3 Fig).

**Fig 6:**
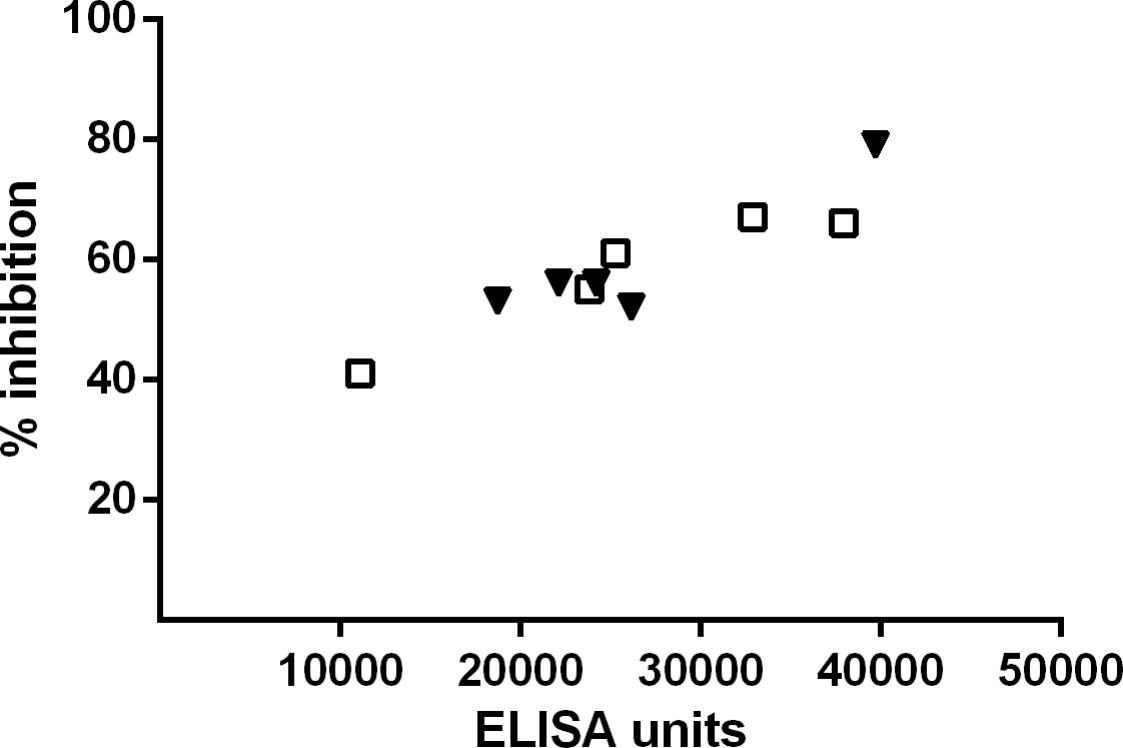
Inhibition of parasite growth *in vitro* with purified IgG from immunized rabbits. Y axis shows % inhibition, and X axis shows the concentration of AMA1-specific antibody used in the assay. Closed triangles represent individual rabbits vaccinated with un-conjugated AMA1. Open squares represents rabbits vaccinated with the AMA1-AMA1 particle.

Antisera from vaccinated mice appear to retain functionality, as evaluated using surrogate functional assays. The growth of parasites in a standardized growth inhibition assay is similarly inhibited by sera from mice vaccinated with AMA1 or the AMA1-AMA1 particle as shown in Fig 6. Sera from mice vaccinated with Pfs25 and Pfs25-EPA were previously shown to inhibit oocyte formation in mosquitoes fed with the sera using a membrane feed assay [29].

## 4. Conclusions

Methods were described for the preparation and characterization of soluble protein nanoparticles from recombinant proteins. The examples that were presented included particles that were prepared from the malaria protein antigens Pfs25, CSP and AMA1 for testing as vaccines. The examples include particles comprised of the antigens alone or the antigens combined with the immunogenic carrier protein EPA. Although the proteins were structurally quite different, they each performed similarly in forming particles, indicating that the described method may have general utility for preparing protein nanoparticles from soluble proteins.

The particles form spontaneously by virtue of the formation of multiple thioether bonds between thiolated and maleimide-modified protein molecules. The Mw and Rh of the particles depend on the concentration and the number of linkers attached, and those parameters can be used to achieve lot to lot consistency. Particles with average molar mass of 500 to 2,000 kDa were targeted for this work because they are large enough to be readily separated from smaller species (monomeric proteins, small conjugates and other reagents or byproducts), yet they can be prepared at reasonable protein concentrations (less than 10 mg/ml). The particles can be readily purified using size separation methods to remove small particles, monomeric proteins, reagents and byproducts. Overall protein yields of greater than 50% after purification are achievable. The described method and scale should be adaptable, with minor modifications, to the preparation of well characterized protein nanoparticles from a wide variety of proteins.

The antigenic properties of the antigenic proteins in the particles appear to be largely unaffected by the process as evidenced by retention of binding to conformation dependent monoclonal antibodies that interfere with biological functions, retention of secondary structure as determined by CD spectroscopy, and ability to generate an antigen-specific antibody responses. Vaccination of mice with nanoparticles of Pfs25H or CSPM3 adsorbed on alum demonstrated that immunogenicity was either elevated or unchanged compared with unmodified antigen. Particles of Pfs25H without carrier generated higher antibody levels than unmodified Pfs25H, whereas particles of CSPM3 without carrier did not appear more immunogenic than unmodified CSPM3. Incorporation of EPA into the particles significantly increased antigenicity for both antigens.

A major advantage of the use of this method to prepare particles for vaccine development, in addition to increasing immunogenicity, is that it can be readily adapted to provide particles for clinical use, that have product profiles closely matching previous preparations used in early phase preclinical testing. Such adaptations may include the use of different techniques or equipment for buffer exchange, liquid handling, column purification, etc. A separate publication describes the development of a cGMP process for scaled-up production of Pfs25H-EPA nanoparticles for clinical use including more extensive biochemical and biophysical characterization [29]. The efficacy of the Pf25-EPA product vaccine is currently being evaluated in clinical trials and has passed safety evaluations (clinicaltrials.gov; ID# NCT01434381) and two additional chemically conjugated transmission blocking vaccines have entered clinical trials (clinicaltrials.gov; ID# NCT01867463, and NCT02334462). It will be important to determine if this strategy of chemical crosslinking to form protein nanoparticles will work for a human vaccine, particularly against malaria.

## Supporting Information

S1 Fig Western blot from 3–8% Tris-acetate, showing binding of conformation dependent AMA1-specific monoclonal antibody 4G2 (see Materials and Methods for details); color generated with alkaline phosphatase-conjugated secondary antibody and BCIP/NBT as substrate. (A) un-conjugated AMA1; (B) AMA1-AMA1.
(TIF)

S2 Fig Western blot from 3–8% Tris-acetate, showing binding of conformation dependent anti-CSP (TSR domain-specific) monoclonal antibody 1G12 (see Materials and Methods for details); color generated with alkaline phosphatase-conjugated secondary antibody and BCIP/NBT as substrate. (A) pre-stained MW markers; (B) CSPM3-EPA.
(TIF)

S3 Fig Antibody levels expressed as ELISA units (extrapolated reciprocal dilution of sera to obtain an OD of 1) in mice vaccinated with AMA1-EPA, AMA1-AMA1 (self-conjugate), and AMA1. Mice were injected SC three times, 14 days apart, with 100 μL of formulation containing 0.5 μg antigen content of the vaccines adsorbed on 160 μg of Alhydrogel. Serum antibody levels were determined two weeks later by ELISA on plates coated with un-modified antigen. Dots represent ELISA units reported for individual mice. Center line represents the geometric mean, and error bars are 95% confidence levels.
(TIF)
