# Supplementary figures and images for: A Method for Producing Protein Nanoparticles with Applications in Vaccines
Source: PLoS One. 2016 Mar 7;11(3):e0138761. doi: 10.1371/journal.pone.0138761 (PMC4780713; doi:10.1371/journal.pone.0138761)

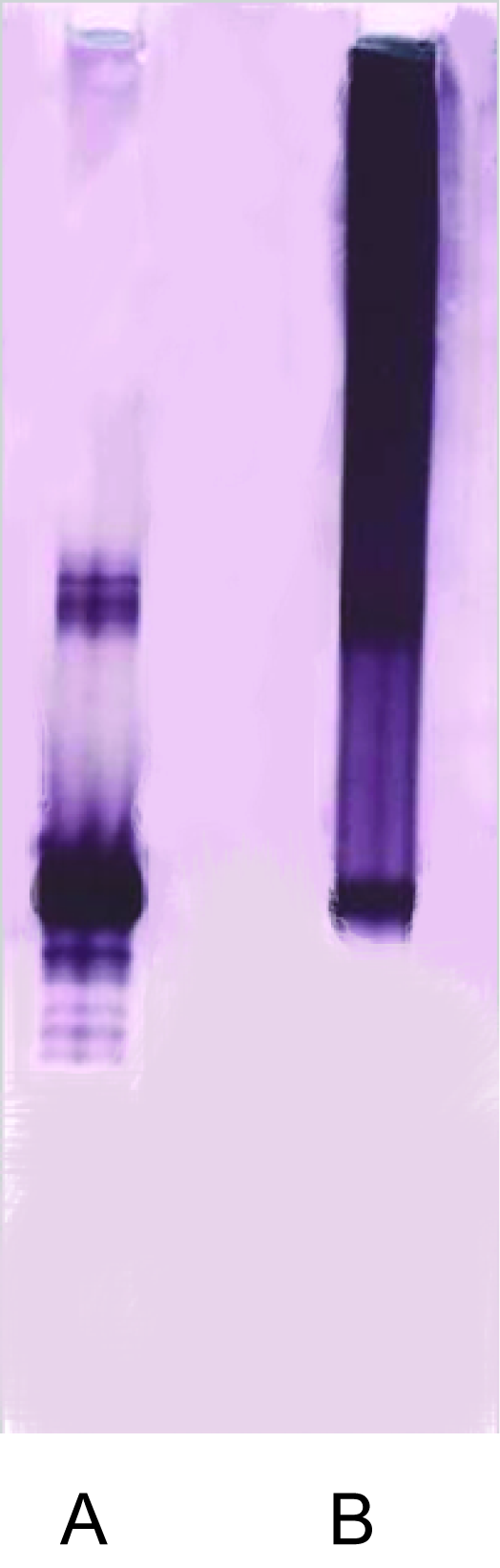

Supplement: S1 Fig — (A) un-conjugated AMA1; (B) AMA1-AMA1. (TIF) [file pone.0138761.s001.tif]

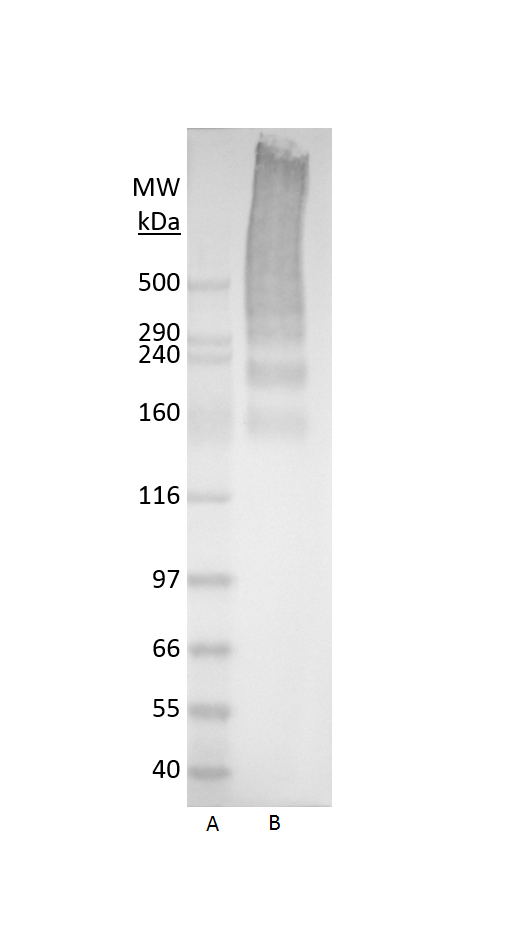

Supplement: S2 Fig — (A) pre-stained MW markers; (B) CSPM3-EPA. (TIF) [file pone.0138761.s002.tif]
